# Investigation of discriminant metabolites in tamoxifen-resistant and choline kinase-alpha-downregulated breast cancer cells using ^1^H-nuclear magnetic resonance spectroscopy

**DOI:** 10.1371/journal.pone.0179773

**Published:** 2017-06-23

**Authors:** Hoe Suk Kim, Lianji Tian, Hyeonjin Kim, Woo Kyung Moon

**Affiliations:** 1 Department of Radiology, Seoul National University Hospital, Jongno-gu, Seoul, Korea; 2 Department of Biomedical Science, College of Medicine Seoul National University, Jongno-gu, Seoul, Korea; University of South Alabama Mitchell Cancer Institute, UNITED STATES

## Abstract

Metabolites linked to changes in choline kinase-α (CK-α) expression and drug resistance, which contribute to survival and autophagy mechanisms, are attractive targets for breast cancer therapies. We previously reported that autophagy played a causative role in driving tamoxifen (TAM) resistance of breast cancer cells (BCCs) and was also promoted by CK-α knockdown, resulting in the survival of TAM-resistant BCCs. There is no comparative study yet about the metabolites resulting from BCCs with TAM-resistance and CK-α knockdown. Therefore, the aim of this study was to explore the discriminant metabolic biomarkers responsible for TAM resistance as well as CK-α expression, which might be linked with autophagy through a protective role. A total of 33 intracellular metabolites, including a range of amino acids, energy metabolism-related molecules and others from cell extracts of the parental cells (MCF-7), TAM-resistant cells (MCF-7/TAM) and CK-α knockdown cells (MCF-7/shCK-α, MCF-7/TAM/shCK-α) were analyzed by proton nuclear magnetic resonance spectroscopy (^1^H-NMRS). Principal component analysis (PCA) and partial least square discriminant analysis (PLS-DA) revealed the existence of differences in the intracellular metabolites to separate the 4 groups: MCF-7 cells, MCF-7/TAM cells, MCF-7-shCK-α cells, and MCF-7/TAM/shCK-α cells. The metabolites with VIP>1 contributed most to the differentiation of the cell groups, and they included fumarate, UA (unknown A), lactate, myo-inositol, glycine, phosphocholine, UE (unknown E), glutamine, formate, and AXP (AMP/ADP/ATP). Our results suggest that these altered metabolites would be promising metabolic biomarkers for a targeted therapeutic strategy in BCCs that exhibit TAM-resistance and aberrant CK-α expression, which triggers a survival and drug resistance mechanism.

## Introduction

Recent advancements in high-throughput technologies, such as nuclear magnetic resonance spectroscopy (NMRS), to obtain cancer-associated metabolites have made a significant contribution to the understanding of cancer properties related to tumor malignancy and response to therapy. Cellular metabolic alterations driven by genetic and environmental factors, have long been known to be associated with the etiology of cancer [1, 2]. Furthermore, metabolites reflect the drug response or resistance of cancer cells, which is currently one of the most important challenges for cancer treatment [3].

Tamoxifen (TAM), which is an antagonist of the estrogen receptor (ER), is one of the most widely used medications for patients with ER-positive breast cancer (BC). Nonetheless, approximately 30% of ER-positive BCs do not respond to the TAM treatment [4]. TAM-resistant breast cancer cells (BCCs) are the primary cause of tumor recurrence and metastasis, and this resistance is one of the major obstacles for treatment of ER-positive BCs [4–6]. TAM treatment results in a higher content of phosphocholine [7, 8], which may therefore be a reflection of the alteration of cellular metabolism, including choline cancer metabolism in TAM-resistant cells. While mechanisms underlying the metabolic alterations associated with TAM-resistance have yet to be characterized, it is well known that increased choline kinase-alpha (CK-α) gives rise to an aberrant choline phospholipid metabolism that is associated with drug resistance as well as the aggressiveness of BCCs [9–12]. Therefore, regulation of aberrant CK-α expression may potentially provide an opportunity to characterize metabolites associated with TAM-resistance, which may be valuable for the advancement of diagnostic and therapeutic strategies for TAM-resistant BCCs [13, 14]. On the other hand, autophagy, which is an essential function for regulating cellular metabolic capabilities, has become an attractive target for cancer therapy because it likely contributes to treatment resistance, dormancy and survival of cancer cells [15–17]. In BCCs, autophagy as a protective and survival mechanism can trigger TAM resistance [15, 18].

Unfortunately, there are still no approved therapies available for clinical use that specifically target TAM-resistant BC. New targeted biomarkers are needed to improve the prognosis for TAM-resistant BC patients. Recently, we reported that CK-α knockdown promoted autophagy induction for survival and dormancy of TAM-resistant and CK-α-knockdown BCCs [19]. Therefore, the discriminant metabolites altered by CK-α knockdown and TAM resistance may allow us to understand the properties of breast cancer cells related to promoting autophagy and TAM resistance. There is no comparative study yet of the analysis of altered metabolites resulting from BCCs with TAM-resistance and CK-α knockdown. In this study, we characterize metabolites that may potentially be associated with TAM-resistance and aberrant CK-α expression using proton nuclear magnetic resonance (^1^H-NMR) in TAM-resistant BCCs.

## Materials and methods

### Cell culture

The ER-positive human breast cancer cell line, MCF-7, was obtained from ATCC (Manassas, VA, USA). The TAM-resistant ER-positive human breast cancer cell line, MCF-7/TAM, was kindly provided by professor Sang-kyu Ye (Department of pharmacology, Seoul national university college of medicine, Seoul, Korea). All these cell lines were cultured in Dulbecco’s Modified Eagle's Media (DMEM) (WelGENE, Daegu, Korea) containing 10% fetal bovine serum, 100 units/ml penicillin, and 100 μg/ml streptomycin. MCF-7/TAM cells were cultured in medium supplemented with 3 μmol/L TAM (Sigma, St. Louis, MO, USA). All cells were incubated at 37°C in a humidified atmosphere of 95% air/5% CO_2_.

### Lentiviral vector infection experiments

The lentiviral vector that carries the transgenes for a CK-α specific shRNA and green fluorescent protein (GFP) was purchased from Thermo Scientific (no. RHS4531). The 293T cells were transfected with a CK-α shRNA lentiviral vector (pLenti-CK-α shRNA), a packing vector (pCMV-dR8.2) and an envelope vector (pCMV-VSVG) using lipofectamine 2000 (Invitrogen, Carlsbad, CA, USA) for lentivirus packaging. Forty-eight hours later, the virus-containing supernatant medium was collected, filtered, and concentrated by ultracentrifugation. In brief, 1x10^5^ MCF-7 and MCF-7/TAM cells were seeded in a six-well plate and infected with lentivirus for 6 hours, and after replacing the culture medium, the cells were incubated for an additional 72 hours. The GFP-expressing cells were separated by a FACSCalibur flow cytometer (BD Biosciences, Franklin Lakes, NJ, USA) equipped with a 530-nm filter (bandwidth, ± 15 nm) and a 585-nm filter (bandwidth, ± 21 nm) then analyzed using CellQuest software (BD Biosciences). The downregulation of CK-α in transduced cells was evaluated by RT-PCR and Western blot. The specific CK-α knockdown cells for MCF-7 and MCF-7/TAM were denoted as MCF-7/shCK-α and MCF-7/TAM/shCK-α, respectively.

### RNA isolation, cDNA synthesis, and real-time RT-PCR

To evaluate the expression levels of CK-α and CK-β in BCCs, real-time RT-PCR was performed. Briefly, total RNA was extracted from cultured cells using TRIzol reagent (Invitrogen, Carlsbad, CA, USA), and the RNA quantity and quality were determined using a NanoDrop spectrophotometer (Thermo Fisher Scientific Inc. Waltham, MA USA). The cDNA was produced using SuperScript II reverse transcriptase (Invitrogen). Real-time PCR assays were run on an ABI PRISM^®^ 7900 using SYBR Green PCR master mix (Applied Biosystems, Foster City, CA, USA). The results were analyzed using the ΔCt method, which reflected the difference in threshold for the target gene relative to the β-actin in each sample. The following primers were used: CK-α, 5′-CTTGGTGATGAGCCTCGGAA-3′, and 5′-AAGTGACCTCTCTGCGAGAA-3′; CK-β, 5′-AGTCTCGGTTCCAGTTCTAC-3′, and 5′-CTTCTGCTCGTTGTTCCTCC-3′; β-actin, 5′-CCAACCGCGAGAAGATGACC-3′ and 5′-GGAGTCCATCACGATGCCAG-3′.

### Western blot

The cells were lysed in RIPA buffer containing a protease inhibitor cocktail (Sigma, St. Louis, MO, USA). The proteins were separated through sodium dodecyl sulfate polyacrylamide gel electrophoresis (SDS-PAGE) and transferred to nitrocellulose membranes. The membranes were blocked with 5% skim milk in Tris-buffered saline and incubated with a primary anti-CK-α antibody (Proteintech Group, Inc., Chicago, IL, USA) and β-actin antibody (Sigma) overnight at 4°C, followed by incubation with horseradish peroxidase-conjugated secondary antibody (Santa Cruz Biotechnology, Santa Cruz, CA, USA) at room temperature for 30 minutes. The blots were developed using enhanced chemiluminescence reagents (Amersham Biosciences, Piscataway, NJ, USA). The relative intensity of the bands observed by Western blotting was analyzed using ImageJ.

### Mitochondrial staining with MitoTracker CMXRos

Functional mitochondria were labeled with the mitochondria-specific red-fluorescent dye Mitotracker Red CMXRos (Molecular Probes, Eugene, OR). In brief, cells were treated with 100 nM Mitotracker Red for 10 min, fixed with 2% paraformaldehyde and counterstained with 4′,6′-diamidino-2-phenylindole. Mitotracker Red fluorescence was detected using a fluorescence microscope (Leica, Wetzlar, Germany). A total of 1.0×10^6^ cells were pelleted and suspended in 1 ml of pre-warmed complete medium with a final concentration of 100 nM Mitotracker Red for 10 min at 37°C. Excess dye was removed with 2 washes in pre-warmed complete medium at 37°C and the mean fluorescence intensity (MFI) value in Mitotracker Red –labeled cells was analyzed using flow cytometry (Becton Dickinson).

### ^1^H-NMR

MCF-7, MCF-7/TAM, MCF-7/shCK-α, and MCF-7/TAM/shCK-α cells were harvested, collected as cell pellets containing 3x10^7^ cells per sample, and stored at -80°C until the onset of ^1^H-NMR data acquisition. In brief, frozen cell pellets were thawed with D_2_O made in PBS, mixed with 1.5 mM sodium 3-(trimethylsilyl)-propionate-2,2,3,3-d4 (TSP; Cambridge Isotope Laboratories, Inc., Andover, MA) as an internal standard, centrifuged to remove precipitates, and then placed immediately on ice until the onset of data acquisition. One-dimensional ^1^H-NMR spectra were acquired on a Bruker Avance 600 system (14.1 T) equipped with 5-mm TXI cryoprobe spectrometers (Bruker BioSpin Corp. Ettlingen, Germany). Spectra were acquired using a CPMG sequence at 20°C±1 with the following sequence parameters: flip angle = 90°/180°, fixed echo time = 1 ms, loop for T2 filter = 20, spectral width = 16 kHz, relaxation delay = 2 s, 32k data points, and 128 scans. NMR spectra were processed using MestReNova (Mestrelab research, Santiago de, Spain) and jMRUI [20]. The time-domain data were apodized with an exponential function (1 Hz) and then Fourier transformed followed by phase- and baseline-correction. The chemical shifts of the peaks were calibrated relative to the TSP signal at 0.00 ppm. The individual metabolites were quantified by estimating the peak areas in the corresponding spectral regions of interest (Table 1), followed by normalization to the measurements for TSP.

**Table 1:** Assignment of metabolites observed by ^1^H-NMR in cancer cell lysates.

Metabolites	^1^H-NMR chemical shift (ppm)
Leucine	1.090–0.810
Isoleucine	1.090–0.810
Valine	1.090–0.810
Alanine	1.499–1.470
Glutarate	1.870–1.850
Acetate	1.957–1.874
N-acetyl-aspartate	2.098–1.968
N-acetyl-amino acid	2.098–1.968
Glutamate	2.379–2.331
Succinate	2.416–2.402
Glutamine	2.496–2.417
Glutathione	2.601–2.525
Creatine	3.052–3.036
Choline	3.215–3.206
Phosphocholine	3.233–3.220
Glycerophosphocholine	3.245–3.233
Taurine	3.456–3.396
Myo-inositol	3.552–3.503
Glycine	3.570–3.552
Lactate	4.105–4.095
Threonine	4.250–4.240
Maleate	6.022–5.957
Fumarate	6.529–6.516
Tyrosine	7.217–7.180
Phenylalanine	7.460–7.409
Histidine	7.888–7.869
Formate	8.443–8.430
AXP	6.022–5.957
UXP	6.022–5.957
NAD	9.161–9.126
unknown A	1.159–1.130
unknown C	5.574–5.538
unknown E	8.620–8.600

### Data analysis

The quantitative analysis of the ^1^H-NMR spectra was performed on 4–6 samples of MCF-7 (n = 4), MCF-7/TAM (n = 4), MCF-7/shCK-α (n = 6) and MCF-7/TAM/shCK-α (n = 5) cells. Given the number of cell groups and ^1^H-NMR-detectable metabolites and the potential correlations among the metabolites, a multivariate analysis was performed using SIMCA (v.13; Umetrics Inc., San Jose, CA). Data were first inspected by performing principal components analysis (PCA), followed by partial least-squares discriminant analysis (PLS-DA). Then, a set of ^1^H-NMR measures were sorted out according to the variable influence on projection (VIP) values as a measure of the relative discriminatory potential of the individual ^1^H-NMR measures [21]. Those ^1^H-NMR measures with VIP>1 were considered to have contributed most to the differentiation of the cell groups, for which further statistical analyses were performed for multiple group comparisons using the Student’s *t*-test and analysis of variance (ANOVA). A *p* value of <0.05 was considered to be statistically significant.

## Results

### Expression of the autophagic maker LC3II in CK-α knockdown and TAM-resistant BCCs

**Fig 1:**
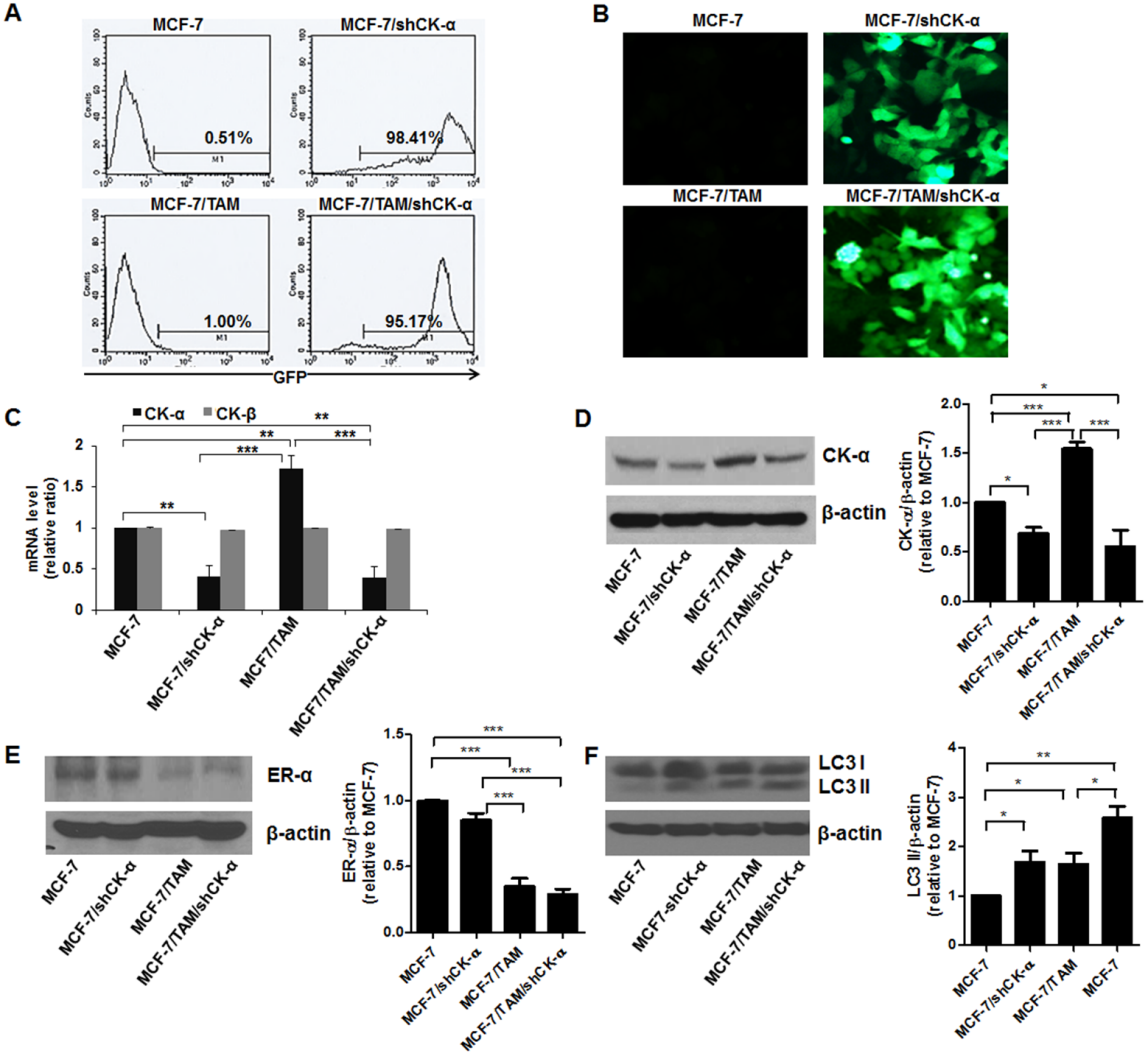
Analysis of choline kinase-α (CK-α), estrogen receptor- and LC3 in CK-α-knockdown cells in MCF-7 and MCF-7/TAM. (A) Flow cytometric analysis of GFP positive cells in MCF-7/shCK-α and MCF-7/TAM/shCK-α transduced with lentivirus containing CK-α shRNA and GFP. (B) Images of GFP expression in MCF-7/shCK-α and MCF-7/TAM/shCK-α. (C) RT-PCR analysis of CK-α and CK-β in MCF-7, MCF-7/TAM, MCF-7/shCK-α and MCF-7/TAM/shCK-α. (D) Western blot analysis of CK-α in MCF-7, MCF-7/TAM, MCF-7/shCK-α and MCF-7/TAM/shCK-α. (E) Western blot analysis of ER-α in MCF-7, MCF-7/TAM, MCF-7/shCK-α and MCF-7/TAM/shCK-α. (F) Western blot analysis of LC3I/II in MCF-7, MCF-7/TAM, MCF-7/shCK-α and MCF-7/TAM/shCK-α. Data are presented as the mean ± standard error. *p<0.05, 0.001<**p<0.05, ***p<0.001.

GFP and CK-α shRNA-transduced cells exhibited over 95% expression of GFP (Fig 1A). A fluorescence microscope showed stable overexpression of GFP in MCF-7/shCK-α and MCF-7/TAM/shCK-α (Fig 1B). CK-α mRNA was significantly higher in MCF-7/TAM (1.72±0.16) relative to MCF-7, and the transduction of shRNA led to a significant and specific downregulation of CK-α mRNA in MCF-7/shCK-α (0.40±0.13) and MCF-7/TAM/shCK-α (0.39±0.13) compared to MCF-7 (***p<0.001, 0.001<**p<0.05, Fig 1C). However, the level of CK-β mRNA was not changed in MCF-7/TAM, MCF-7/shCK-α and MCF-7/TAM/shCK-α relative to MCF-7. As shown in Fig 1D, the levels of CK-α protein assessed by Western blot were higher in MCF-7/TAM (1.55±0.07) and decreased in MCF-7/shCK-α (0.68±0.06) and MCF-7/TAM/shCK-α (0.55±0.17) compared to MCF-7 (*p<0.05, 0.001<**p<0.05, ***>*p*). As expected, ER-α expression decreased in MCF-7/TAM (0.35±0.12) and MCF-7/TAM/shCK-α (0.28±0.08) (***p<0.001, Fig 1E). LC3II was used as a reliable autophagosome marker for monitoring autophagy and was increased in MCF-7/shCK-α (1.66±0.65), MCF-7/TAM (1.64±0.44) and MCF-7/TAM**/**shCK-α (2.48±0.31) compared to the parental MCF-7 (*p<0.05, 0.001<**p<0.05, Fig 1F).

Only a few caspase-3-positive cells were observed in MCF-7/shCK-α. However, shRNA-mediated partial CK-α knockdown was not sufficient to render apoptotic cell death and decreased cell proliferation activity in MCF-7/TAM/shCK-α (S1 Fig). We evaluated the mitochondrial dysfunction in cells loaded with MitoTracker CMXRos to examine changes in either mitochondrial mass, membrane potential, or both. Fluorescence microscopy of MCF-7/shCK-α and MCF-7/TAM/shCK-α revealed that selective mitochondria staining using MitoTracker CMXRos decreased through CK-α knockdown (S2 Fig). The mean fluorescence intensity (MIF) of MitoTracker CMXRos was significantly lower in MCF-7/shCK-α and MCF-7/TAM/shCK-α, and the intensity was significantly higher in MCF-7/TAM, which suggests there were changes in mitochondrial mass and mitochondrial membrane potential (S2 Fig).

### Analysis of metabolites by ^1^H-NMR in CK-α knockdown and TAM-resistant BCCs

The ^1^H-NMR spectra of the samples from MCF-7 (MC), MCF-7/TAM (MT), MCF-7/shCK-α (Msh) and MCF-7/TAM/shCK-α (MTsh) showed a wide variety of metabolite resonances. A total of 30 metabolites were identified, which included amino acids, energy metabolism-related molecules (e.g., pyruvate, succinate, lactate, creatine), and others (cholines, amines, amides, nucleotides, etc.). There were also 3 unidentified resonances (Table 1).

**Fig 2:**
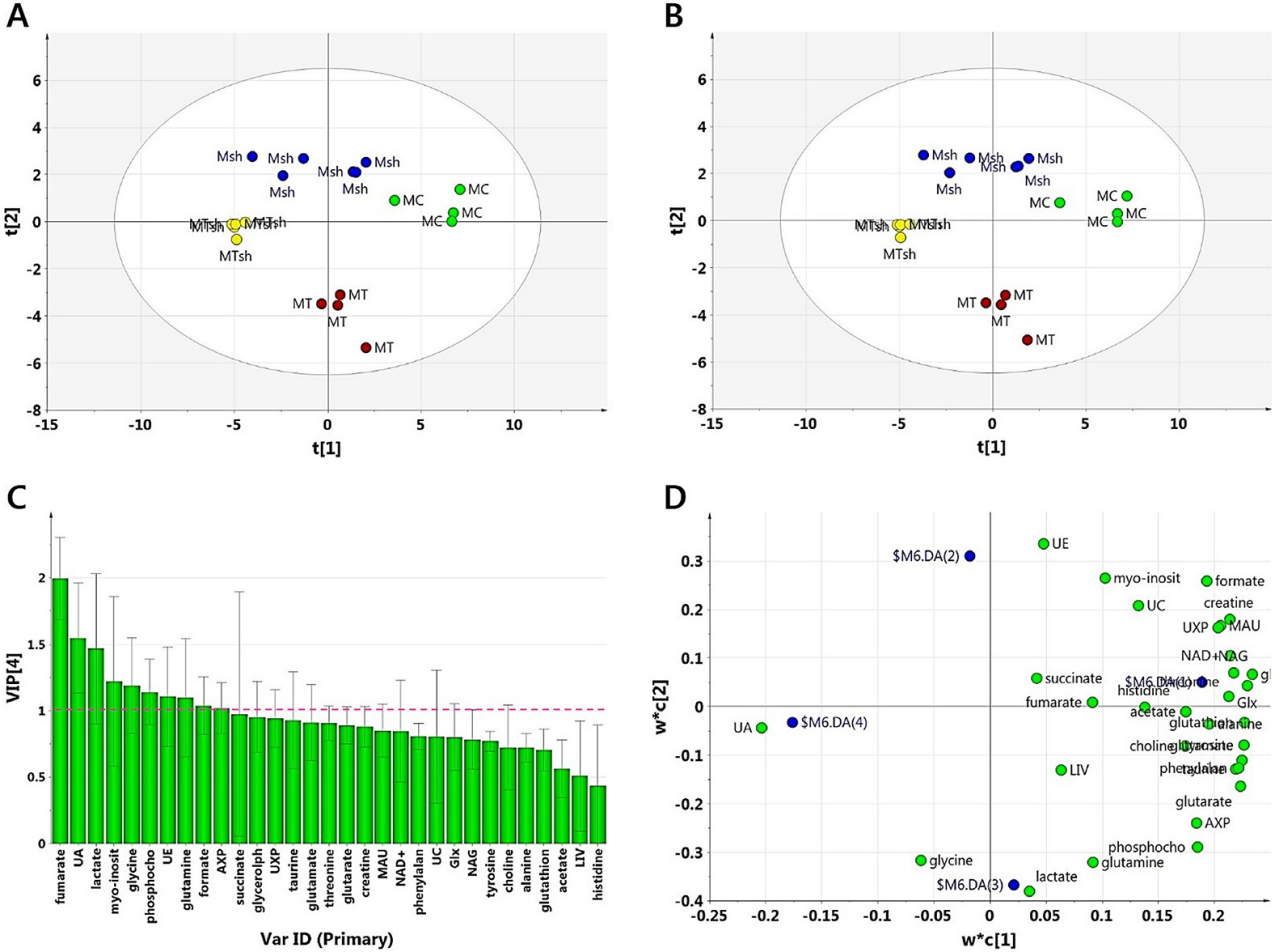
PCA and PLS-DA for MCF-7, MCF-7/shCK-α, MCF-7/TAM and MCF-7/TAM/shCK-α groups. (A) PCA for the inspection and overview of the data set. (B) Score plot for the investigation of group differentiation among MCF-7 (MC, n = 4, green), MCF-7/shCK-α (Msh, n = 6, blue), MCF-7/TAM (MT, n = 4, red), and MCF-7/TMA/shCK-α (MTsh, n = 5, yellow) cell groups. (C) VIP values as a measure of the discriminatory potential of the individual metabolites in the group differentiation. (UA: unknown resonance A, lactate, UE: unknown resonance E, glutamine, formate, AXP: AMP/ADP/ATP, UXP: UMP/UDP/UTP, MAU: maleate/AXP/UXP, UC: unknown resonance C, NAG: N-acetyl-aspartate/-amino acid/glutamate, Glx: glutamate/glutamine, LIV: leucine/isoleucine/valine). (D) Loading plot showing the relative contribution of the metabolites to the scores in the score plot. By matching the positions in the score plot and loading plot, the correlations between the individual metabolites and the cell groups were determined.

In the PCA analysis, the MCF-7 (MC), MCF-7/TAM (MT), MCF-7/shCK-α (Msh) and MCF-7/TAM/shCK-α (MTsh) groups were well separated from each other without potential outliers (Fig 2A). In the PLS-DA analysis, the cell groups were well differentiated among each other (Fig 2B). The score plot (Fig 2B) also showed effective differentiation of the cell types according to TAM-resistance ((MCF-7(MC) and MCF-7/shCK-α(Msh) vs. MCF-7/TAM(MT) and MCF-7/TAM/shCK-α(MTsh)) and CK-α knockdown ((MCF-7 (MC) and MCF-7/TAM(MT) vs. MCF-7/shCK-α(Msh) and MCF-7/TAM/shCK-α(MTsh)). According to the loading plot (Fig 2D) in combination with the score plot (Fig 2B), the majority of the metabolites tended to be higher in MCF-7 (MC) than in the rest of the cell groups. The relative contributions of the metabolites to the differentiation of the cell groups are shown in order in Fig 2C. The metabolites with VIP>1 were fumarate, UA (unknown A), lactate, myo-inositol, glycine, phosphocholine, UE (unknown E), glutamine, formate, and AXP (AMP/ADP/ATP).

### Key metabolites altered by CK-α knockdown in TAM-resistant BCCs

**Fig 3:**
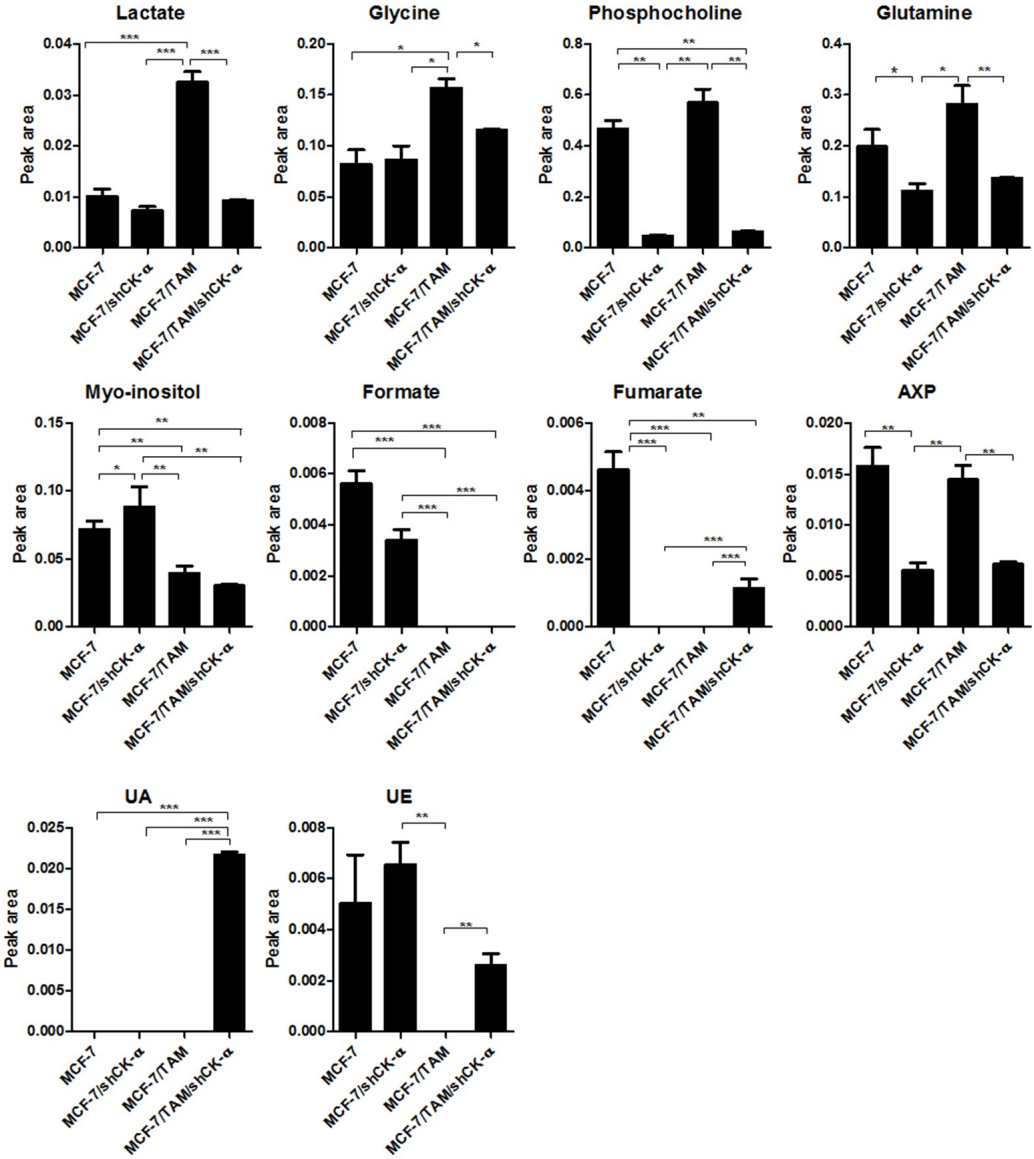
Comparison of the concentrations of the metabolites with VIP>1 among the MCF-7, MCF-7/shCK-α, MCF-7/TAM, and MCF-7/TAM/shCK-α cell groups. Increased lactate, glycine, phosphocholine, glutamine, and succinate in MCF-7/TAM relative to the other cell groups. Increased myo-inositol in MCF-7/shCK-α relative to the other cells. Non-detectable formate in MCF-7/TAM and MCF-7/TAM/shCK-α. Non-detectable fumarate in 7/shCK-α and MCF-7/TAM and MCF-7/TAM/shCK-α. Decreased AXP in MCF-7/shCK-α cells and MCF-7/TAM/shCK-α relative to MCF-7 and MCF-7/TAM. The spectra were normalized against total intensity and averaged over the samples in MCF-7, MCF-7/shCK-α, MCF-7/TAM and MCF-7/TAM/shCK-α. All values are presented as the mean ± standard error. *p<0.05, ** 0.001*<p*<0.05, *** *p*<0.001.

Fig 3 shows the results of the t-test between the cell groups for those metabolites (fumarate, UA, lactate, myo-inositol, glycine, phosphocholine, UE, glutamine, formate, and AXP) with VIP>1. There is a trend towards high concentrations of lactate, glycine, phosphocholine, and glutamine in the MCF-7/TAM with respect to the other three cell groups. The MCF-7/shCK-α had significantly higher myo-inositol concentrations than the other cell groups. The concentrations of formate were negligible in the TAM-resistant cell groups (MCF-7/TAM and MCF-7/TAM/shCK-α). Fumarate was detected only in MCF-7 and MCF-7/TAM/shCK-α. Phosphocholine, glutamine and AXP were remarkably decreased in the CK-α knockdown cell group (MCF-7/shCK-α and MCF-7/TAM/shCK-α). Unknown A was detected only in MCF-7/TAM/shCK-α. Unknown E was not detected in MCF-7/TAM. The results of the t-test for the rest of the metabolites are shown in S1 Fig. Overall, CK-α knockdown and/or TAM-resistance led to decreased metabolite concentrations, including choline-related metabolites (S3 Fig).

## Discussion

Although it has anti-estrogen activity, TAM is widely used to treat ER-positive breast tumors as an adjuvant therapy for early-stage hormone-sensitive breast cancer or first line therapy for metastatic hormone-sensitive breast cancer, and as many as 30% of patients can be refractory to TAM and acquire resistance to TAM along with the loss of ER-α [6, 22–24]. CK-α is one of the targets of therapeutic strategies because it is a key enzyme in choline metabolism and might contribute to TAM resistance [11, 19]. In addition, the metabolic survival-promotion function of autophagy, which is activated in drug resistant BCCs, is also one of the therapeutic targets [19]. Our recent study reported a potential connection between autophagy and CK-α in the mechanisms driving TAM resistance in ER+ BCCs. Interestingly, TAM-resistant and/or CK-α-knockdown BCCs (MCF-7/TAM and MCF-7/TAM/shCK-α) exhibited notable induction of autophagy through accumulation of LC3-II and P62 as well as the suppression of AKT, ERK and mTOR, which can contribute to the protective and survival mechanisms of MCF-7/TAM and MCF-/TAM/shCK-α cells [19]. According to the study reported by Sanchez-Lopez *et al* [25], the pharmacological inhibition of CK-α by MN58b and RSM932A changes CK-α protein folding and leads to apoptosis via CHOP-mediated ER stress in cancer cells, including MCF-7, but partial genetic inhibition of CK-α by small interfering RNA (siRNA) does not induce apoptosis. The potent downregulation of endogenous CK-α protein using siRNA in breast cancer cells (MDA-MB-231, MDA-MB-468) and cervical cancer cells (HeLa) reduces proliferation, and results in significant cell death through apoptosis [12, 26, 27]. We rarely observed few caspase-3-stained cells, indicating that there is an apoptotic response in MCF-7/shCK-α but not MCF-7/TAM/shCK-α as well as a reduction of proliferation activity in MCF-7/TAM/shCK-α, suggesting that there is partial downregulation (approximately 30%) of the CK-α proteins in our shRNA system that is not sufficient to render apoptotic cell death but reduces proliferation activity in MCF-7/TAM/shCK-α. The partial knockdown of CK-α protein in our study may limit the reproducibility of previous studies. In addition, these discrepancies with the many previous reports would be due to distinct pharmacokinetic or target selectivity of pharmacological inhibitors as well as different knockdown efficiency of the siRNA or shRNA. When CK-α is inhibited either genetically (shRNA) or pharmacologically (CK37) in our previous study [19], shRNA and CK37 increased the autophagosomal marker LC3-II expression, but rendered differential effects on the expression level of p62, a marker of autophagic flux as shRNA, which suggest that genetic or pharmacological inhibition of CK-α can perturb a biological and metabolic system in different ways. Besides being a competitive CK inhibitor, CK37 suppresses choline uptake [28]. In general, different cellular responses can be triggered by concentration- and time-dependent pharmacokinetics of CK37. Therefore, pharmacological inhibitor should be used with caution. For this reason, the metabolic analysis of CK37-treated cells was not performed in this study. In our study, the lack of correlation between the levels of mRNA and proteins of CK-α was observed in CK-α knockdown cells. This is because protein levels are generally affected by many steps in their synthesis, stability and degradation [29]; cells can control the rates of degradation and synthesis of proteins depending on a number of different conditions, even for those proteins with similar functions. We speculate that the lack of a strong downregulation of the CK-α protein levels in CK-α knockdown cells may be associated with the steps of high stability or low degradation.

We designed the study to depict metabolic differences based on TAM resistance and CK-α expression linked with protective autophagy, which could potentially provide a direction toward targets for validation studies and the development of therapeutics in ER+ BC patients. To the best of our knowledge, this is the first study to apply ^1^H-NMR to identify altered metabolites in the total lysate of TAM-resistant and/or CK-α-knockdown BCCs linked with TAM resistance as well as protective autophagy for use as predictors of the hormone and CK-α gene therapy. In the present study, we quantified a total of 33 metabolites (including 3 unknown resonances) in the MCF-7, MCF-7/shCK-α, MCF-7/TAM and MCF-7/TAM/shCK-α cells. In the subsequent multivariate analysis, a statistical model was constructed that effectively differentiated cell types according to TAM-resistance and CK-α expression. The metabolites that contributed most to differentiation were found to be fumarate, UA, lactate, myo-inositol, glycine, phosphocholine, UE, glutamine, formate, and AXP. Increased glycolysis has been linked to drug resistance through increased lactate production [30]. It was also reported very recently that lactate is critical for sustaining protective autophagy in cancer cells, including ovarian carcinoma cells, glioblastoma cells and gastric cancer cells [31, 32]. In addition, elevated lactate is associated with drug resistance and stemness of BCCs, which drives recurrence, metastasis and poor clinical outcomes in BC patients [30, 32, 33]. Glycine provides the essential precursors for the synthesis of proteins, nucleic acids, and lipids, and it is a crucial metabolite for cancer cell proliferation and growth [34–36]. Glutamine participates as a nutrient in energy formation, redox homeostasis, and macromolecular synthesis and influences the suppression/induction of autophagy through a complex mechanism [37, 38]. High expression and activity of CK-α in BCCs elevates phosphocholine levels, which serves as a biomarker, thus reflecting breast cancer progression, especially in cases of drug resistance [9, 10, 22, 39, 40]. As noted in the results above, the MCF-7/TAM cells exhibiting TAM resistance and protective autophagy had high levels of lactate, glycine, glutamine, and phosphocholine, whereas CK-α knockdown reduced the levels of these metabolites, suggesting that these metabolites could be regulated by CK-α-mediated mechanisms and could serve as biomolecules and energy sources to overcome TAM insult and activate protective autophagy of damaged products. The level of myo-inositol was reported to be higher in MCF-7 and BT-474 cells (luminal A type of breast cancer cell line) than in MDA-MB-468 and MDA-MB-231 cells (triple negative breast cancer cell line), and there was also an increase in paclitaxel [41].

The CK defect causes a decreased phosphatidylcholine (PtdCho) level in the mitochondrial membrane, leading to mitochondrial dysfunction and degradation by autophagy through a process called mitophagy [27, 42]. The measurement of PtdCho requires separate preparation of cells, for instance, cells dissolved in methanol-chloroform solvent, for precise quantification of PtdCho levels from 1H-NMR spectra [43, 44], which was not available in our study. For this reason, we did not attempt to quantify PtdCho in our samples dissolved in D_2_O, which is one of the limitations of our study. Given its importance in the metabolism of BCCs [43, 45], the measurement of PtdCho would have provided additional information in the interpretation of our data. For instance, it might have corroborated the relationships of PtdCho with mitochondrial dysfunction, especially autophagy and drug resistance in both CK-α knockdown cells and TAM-resistant cells.

Interestingly, we found that CK-α knockdown in ER+BCCs led to a decrease in MitoTracker CMXRos-stained mitochondria and the MIF of MitoTracker CMXRos, which indicates that there is mitochondrial dysfunction. In cancer patients, cancer cells with “healthy mitochondria” are actually more resistant to conventional therapies [46]. The treatment with TAM can induce the aggregation of mitochondria and mitochondrial-mediated apoptosis in BCCs [47], but mitochondrial function is improved in TAM-resistant BCCs [48]. In accordance with these findings, we observed that MCF-7/TAM cells exhibited higher levels in mitochondria staining and MIF of MitoTracker CMXRos compared to parent MCF-7 cells and MCF-7/shCK-α and MCF-7/TAM/shCK-α exhibited a loss of mitochondria compared to MCF-7 and MCF-7/TAM. According to a previous study by Rodríguez-González *et al*. [49], a strong CK-α inhibition by MN58b in cervical cancer cells induces cytochromc c release, followed by a loss of mitochondrial potential, which are associated with mitochondria-mediated apoptosis. Our study shows that a partial knockdown of CK-α by shRNA in breast cancer cells preferentially induced mitochondria-mediated autophagy. The different outcomes reported by Rodríguez-González *et*. *al* may likely be due to stronger inhibition of CK-α activity in their study. Our findings suggest that understanding mitochondrial dysfunction (mitochondria-mediated autophagy or apoptosis) may be the key to unlocking new anticancer therapies and preventing the onset of drug resistance in cancer patients. Additionally, mitochondrial metabolism may be a key target for designing novel anticancer therapies.

The depletion of myo-inositol induces protective autophagy in human cells by influencing both ER and mitochondria [50, 51]. Therefore, the low levels of myo-inositol in TAM-resistant cells (MCF-7/TAM and MCF-7/TAM/shCK-α) might have contributed to the induction of protective autophagy. Some cancer cells exhibit a high rate of formate release when growing in a standard culture medium, and a recent study has shown that electron transport chain dysfunction leads to a reduction in mitochondrial formate production from serine [38]. An excess amount of intracellular fumarate due to mutation or loss of fumarate hydratase is detected in the majority of primary tumors and metastases, and the involved mechanisms are potentially associated with treatment resistance [52]. Formate and fumarate also have critical roles in regulating epigenetic changes and maintaining cellular redox homeostasis [53]. Of note, formate and fumarate were remarkably decreased by CK-α knockdown and were hardly detected in the MCF-7/TAM cells. Based on the above reports, undetectable formate and fumarate in MCF-7/TAM cells might be associated with excessive activation of fumarate hydratase and dysfunction of the mitochondrial electron transport chain. These results suggest that the fumarate hydratase and mitochondrial electron transport chain could also be the target for TAM-resistance and protective autophagy in ER+ BC patients.

In conclusion, the discriminant metabolites found in TAM-resistant and/or CK-α knockdown BCCs can be used as metabolic markers to predict TAM resistance and aberrant CK-α expression and may represent changes in the metabolic activity of signaling pathways underlying the protective autophagy triggered by TAM resistance and CK-α knockdown. Our findings may provide further insight into the different therapeutic responses of BCCs and additional information about current diagnostic and therapeutic assessments of TAM-resistant BCCs. Further studies on the blood samples or needle biopsies from TAM-resistant BC patients would be required to validate the potential clinical applicability of the metabolic biomarkers found in the BCCs with TAM resistance and aberrant CK-α expression.

## Supporting information

S1 Fig Caspase-3 and Ki-67 staining and cell cycle analysis in MCF-7, MCF-7/shCK-α, MCF-7/TAM and MCF-7/TAM/shCK-α. (A) Immunostaining of activated caspase-3, which indicates apoptotic response. A small amount of caspase-3 staining (white arrow) was observed only in MCF-7/shCK-α. (B) Immunostaining of Ki-67, which indicates proliferative activity. The Ki-67-stained cell population (red arrow) was decreased in MCF-7/shCK-α, MCF-7/TAM and MCF-7/TAM/shCK-α. (C) Cell cycle analysis using flow cytometry with propidium iodide. A decrease in the S phase population and an increase in the G0/G1 phase population were observed in MCF-7/TAM and MCF-7/TAM/shCK-α relative to MCF-7 and MCF-7/shCK-α. An intriguing finding in MCF-7/TAM/shCK-α was a further increase in the G0/G1 phase population and a decrease in the S phase population compared to MCF-7/TAM.
(TIF)

S2 Fig Analysis of mitochondria in MCF-7, MCF-7/shCK-α, MCF-7/TAM and MCF-7/TAM/shCK-α using the mitochondrial dye MitoTracker CMXRos. (A) Fluorescence microscopy and (B) flow cytometry analysis of mitochondrial dye uptake. The MitoTracker CMXRos-stained mitochondria decreased in MCF-7/shCK-α. The mean fluorescence intensity (MIF) of MitoTracker CMXRos was significantly lower in MCF-7/shCK-α and MCF-7/TAM/shCK-α, and it was significantly higher in MCF-7/TAM, which suggests that there is a change in mitochondrial mass or mitochondrial membrane potential compared to the parent MCF-7. Scale bar, 20 μm. All values are presented as the mean ± standard error. * *p*<0.05, ** 0.05<*p*<0.001, *** *p*<0.001.
(TIF)

S3 Fig Statistical significance of metabolites analyzed in MCF-7, MCF-7/shCK-α, MCF-7/TAM and MCF-7/TAM/shCK-α. The peak areas were normalized to total signal and averaged across the samples for each individual groups of MCF-7, MCF-7/shCK-α, MCF-7/TAM and MCF-7/TAM/shCK-α. All values are presented as the mean ± standard error. * *p*<0.05, ** 0.05<*p*<0.001, *** *p*<0.001.
(TIF)
