# Supplementary figures and images for: Investigation of discriminant metabolites in tamoxifen-resistant and choline kinase-alpha-downregulated breast cancer cells using 1H-nuclear magnetic resonance spectroscopy
Source: PLoS One. 2017 Jun 23;12(6):e0179773. doi: 10.1371/journal.pone.0179773 (PMC5482454; doi:10.1371/journal.pone.0179773)

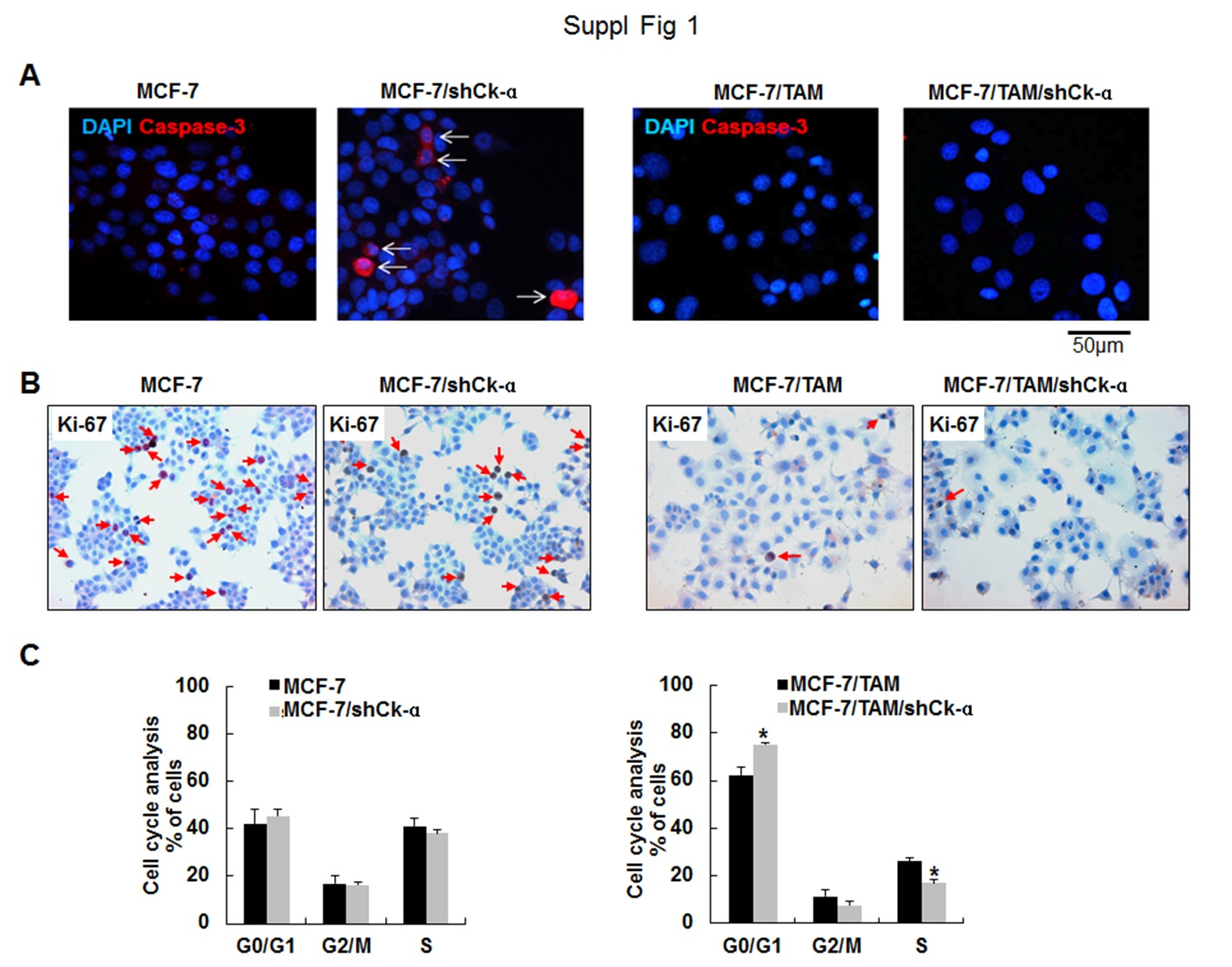

Supplement: S1 Fig — (A) Immunostaining of activated caspase-3, which indicates apoptotic response. A small amount of caspase-3 staining (white arrow) was observed only in MCF-7/shCK-α. (B) Immunostaining of Ki-67, which indicates proliferative activity. The Ki-67-stained cell population (red arrow) was decreased in MCF-7/shCK-α, MCF-7/TAM and MCF-7/TAM/shCK-α. (C) Cell cycle analysis using flow cytometry with propidium iodide. A decrease in the S phase population and an increase in the G0/G1 phase population were observed in MCF-7/TAM and MCF-7/TAM/shCK-α relative to MCF-7 and MCF-7/shCK-α. An intriguing finding in MCF-7/TAM/shCK-α was a further increase in the G0/G1 phase population and a decrease in the S phase population compared to MCF-7/TAM. (TIF) [file pone.0179773.s001.tif]

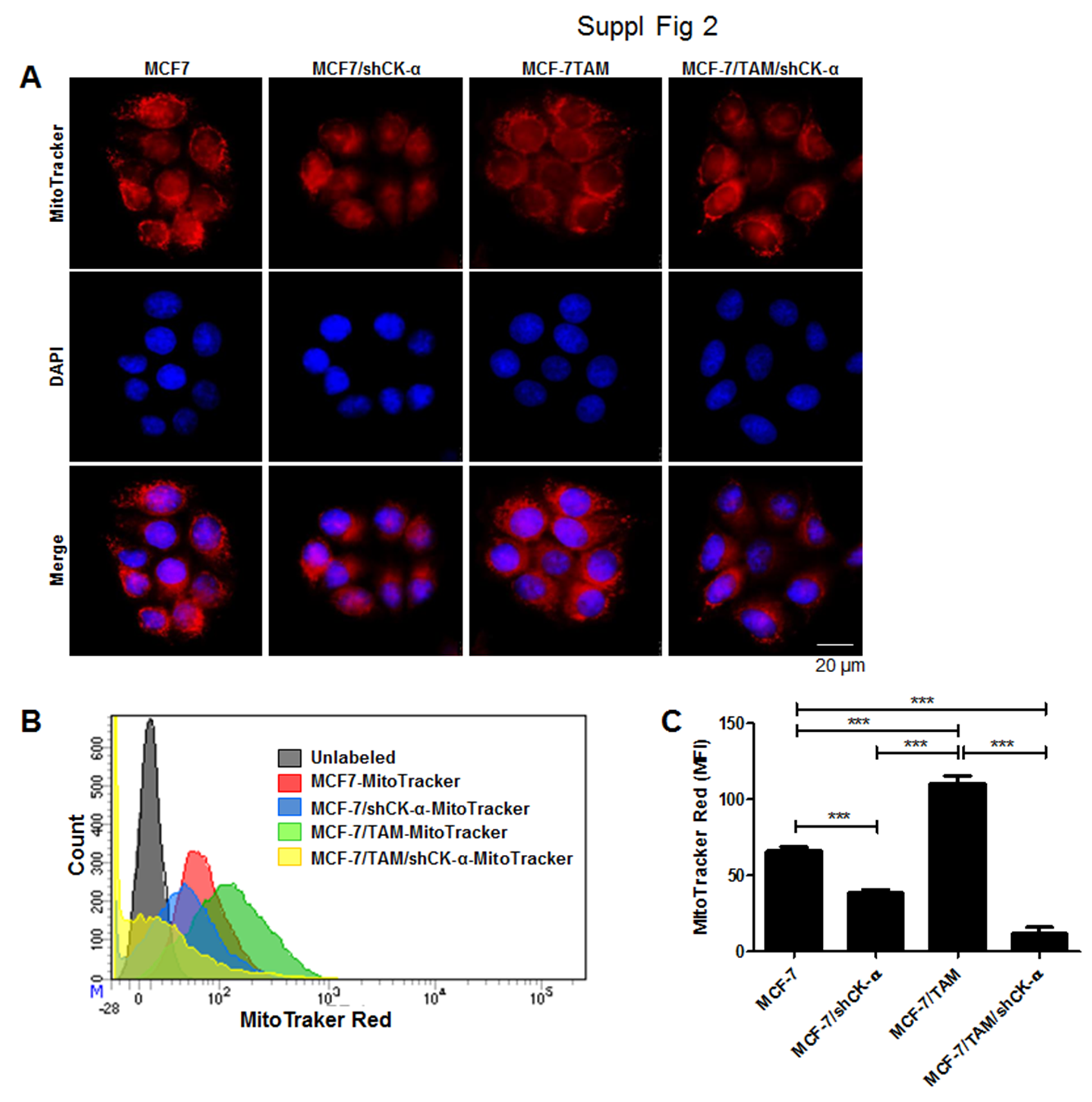

Supplement: S2 Fig — (A) Fluorescence microscopy and (B) flow cytometry analysis of mitochondrial dye uptake. The MitoTracker CMXRos-stained mitochondria decreased in MCF-7/shCK-α. The mean fluorescence intensity (MIF) of MitoTracker CMXRos was significantly lower in MCF-7/shCK-α and MCF-7/TAM/shCK-α, and it was significantly higher in MCF-7/TAM, which suggests that there is a change in mitochondrial mass or mitochondrial membrane potential compared to the parent MCF-7. Scale bar, 20 μm. All values are presented as the mean ± standard error. * p<0.05, ** 0.05<p<0.001, *** p<0.001. (TIF) [file pone.0179773.s002.tif]

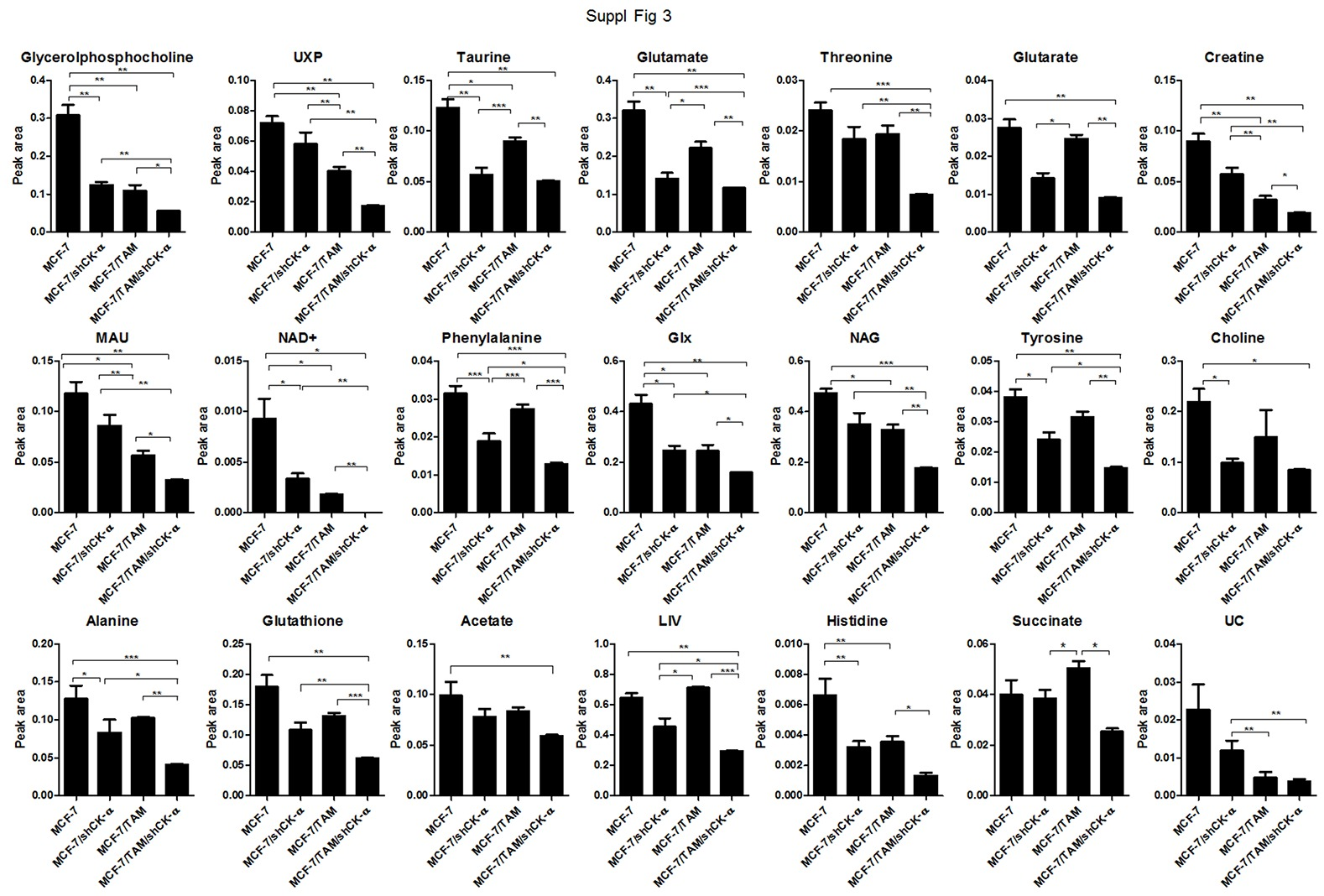

Supplement: S3 Fig — The peak areas were normalized to total signal and averaged across the samples for each individual groups of MCF-7, MCF-7/shCK-α, MCF-7/TAM and MCF-7/TAM/shCK-α. All values are presented as the mean ± standard error. * p<0.05, ** 0.05<p<0.001, *** p<0.001. (TIF) [file pone.0179773.s003.tif]
